# Boosting antioxidative polyphenols extraction efficiency via nano sized pomegranate peel particles

**DOI:** 10.1038/s41598-025-99051-3

**Published:** 2025-05-07

**Authors:** Eman A. M. Mahmoud, Yasser F. M. Kishk, Ibrahim Khalifa, Abdel Fattah A. Abdel Fattah, Samar M. Mahdy

**Affiliations:** 1 Department of Food Science and Technology, Faculty of Agriculture, Al-Azhar University, Cairo, Egypt; 2 Department of Food Science, Faculty of Agriculture, Ain Shams University, Cairo, Egypt; 3 Food Technology Department, Faculty of Agriculture, Benha University, Moshtohor, Qaliuobia 13736 Egypt

**Keywords:** Pomegranate, Polyphenols, Nanoparticles, Optimization, Antioxidants, Biological techniques, Environmental sciences

## Abstract

We aimed at maximizing the utilization of pomegranate peels as a phenolics-rich agro-waste and increasing their extractability. The five factors of composite design, namely methanol concentrations (C), soaking time (t), temperatures (T), powder-solvent ratio (R), and nanoparticle diameter (D) were studied. Pomegranate peel powder (PPP) and its nano-fractions (PPPN1 and PPPN2) were then prepared and characterized. The particle size, surface morphology, total phenolics, chemical structure, phenolic acids profile, radical scavenging (RSA), reducing power (RP), and ferric reducing antioxidant power (FRAP) assays were determined. PPPN1 exhibited larger particle sizes (347 nm) compared to PPPN2 (112 nm) with a spherical surface morphology. PPPN2 exhibited the highest total phenolics extractability (344 mg GAE g^−1^) which was proved by Fourier-transform infrared spectra. It had also the high total free, conjugated, and bound phenolic values of 59.64, 18.44, and 111.18 mg g^−1^, orderly. The quintic polynomial regression model predicted a phenolics yield of 406 mg GAE g^−1^, achieved at 75% C, 45 min, 80 °C, 16.7% R, and 112 nm D. PPPN2 extract exhibited high RSA, RP, and FRAP values compared to butylated hydroxytoluene. This work enhanced pomegranate peel phenolic extraction, highlighting their potential for food manufacture and requiring additional investigation.

## Introduction

Pomegranate (*Punica granatum* L.) fruit belongs to the Punicaceae family and is widely consumed globally for its appreciable taste^1^. Pomegranate peel constitutes ~ 60% of the fruit’s weight as an agricultural waste^2^. These peels contain a significant amount of phenolics compared to pulp^3^. Pomegranate peel is, especially, rich source of phenolic acids such as gallic, ellagic, and caffeic acids^4^. The pomegranate peel extract is a rich in beneficial bioactive polyphenols^5^. The high phenolic content of pomegranate peel contributes to its health benefits, antioxidant, antimicrobial, antihypertensive, antilipidemic, and antidiabetic properties^6^. Using pomegranate peel has both economic and environmental benefits, as it reduces organic pollutants. Increasing the efficiency of extracting polyphenols from agricultural waste allows us to manufacture high-value, high-quality chemicals^7^. Because of their chemical qualities, the macromolecules present in pomegranate peel and its extract have been proposed as natural alternatives for manufactured nutraceuticals, food additives, and chemo-preventive agents^8^.

Polyphenols are widely distributed in the plant kingdom, with numerous studies demonstrating their antioxidant capabilities^9^. Polyphenols consistently attract the attention of researchers exploring their antioxidant, antimicrobial, and health-promoting functions^10^. Thus, instead of considering pomegranate peel as waste, it should be recognized as a valuable byproduct^8,11^. To prevent the rancidity of fats and oils, synthetic antioxidants such as butylated hydroxyanisole, butylated hydroxytoluene, and tertiary butyl hydroquinone have been used. However, concerns regarding the toxic nature of synthetic antioxidants and increasing trend of natural antioxidants usage are present nowadays^12^.

Meanwhile, nanoparticles offer distinct advantages due to their high surface-to-volume ratio^13^, making them suitable for various applications in the realm of food control^14^. Given the potential of polyphenols to reduce the occurrences of cancers, diabetes, and coronary heart disease, they have garnered significant interest in the food industry. Additionally, nanoparticles are designed to facilitate the direct delivery of these phenolic phytochemicals into the human body^15^. Researchers employed various techniques, such as ultrasound waves^16^, and a variety of solvents^17^, to enhance the extraction of polyphenols from powdered pomegranate peels.

Hence, the objective of this study was to optimize the isolation process of polyphenols from pomegranate peels. Independent variables, i.e., methanol concentrations, soaking time, temperatures, powder-solvent ratio, and nanoparticle diameters were used to define a range of variables using the 3D-response surface method. The resulting extract with a high phenolic yield was evaluated for its antioxidant properties compared to the synthetic antioxidant butylated hydroxytoluene (BHT). Overall, the novelty of this work lies in the comprehensive, multi-variable optimization (methanol concentration, time, temperature, powder-solvent ratio, and nanoparticle diameter) applied to pomegranate peel polyphenol extraction; a combination not previously explored in this context. By employing 3D-response surface methodology, we systematically identified synergistic interactions among these parameters, achieving a high phenolic yield with enhanced antioxidant efficacy. This approach advances sustainable valorization of agro-waste by refining extraction efficiency while demonstrating the potential of natural antioxidants to replace synthetics.

## Materials and methods

The pomegranate (*Punica granatum* L.) fruits were acquired from a local market in Cairo, Egypt. All the chemicals and reagents utilized in this study were procured from Sigma-Aldrich (St. Louis, MO, USA). MillQ-H_2_O (Millipore, USA) was also used herein.

### Preparation and characterization of PPPN1 and PPPN2

The pomegranate peels were dehydrated for one week at room temperature (23–25 °C). Subsequently, the dried peels were ground to produce PPP, which was then sieved through a 30-mesh sieve. The preparation of pomegranate nanoparticles involved using PPP in a Planetary Ball Mill (Model: PQ-N2, Gear Drive 4-station, 220 V, Retsch, Germany), following a related method^3^. The milling process took place at room temperature for two different durations: 60 and 120 min. Based on the milling time, the resulting nano-pomegranate powders were classified as PPPN1 and PPPN2, respectively.

The particle size of PPPN1 and PPPN2 was determined using a related method^18^. Nano-suspensions of PPPN1 and PPPN2 were prepared by suspending 20 mg of each particle in 4 mL of ddH_2_O with the addition of 0.5 mL of dimethyl sulfoxide. The suspensions were homogenized by stirring for 30 min at 23–25 °C and then centrifuged for 15 min in 1860 ×g. Serial dilutions of the supernatant were prepared using ddH_2_O to obtain various concentrations. The measurements were conducted after a 5-min equilibration period at 25 °C, based on the electrophoretic mobility under an electric field. The particle size measurements were performed using a Zetasizer (Malvern, Model: Zetasizer Nano Series, Nano ZS, UK) with a dynamic laser scattering angle of 173°. The size range was between 0.6 and 6000 nm, and the zeta potential ranged from −200 to 200 mV. The average of 16 runs was obtained with a duration time of 10 s.

The particle size and surface morphology of PPPN1 and PPPN2 particles were analyzed using a JEOL JX 1230 transmission electron microscope (TEM) equipped with a microanalyzer probe from Japan.

Fourier-transform infrared spectroscopy (FTIR) was employed to examine the chemical structure of the components in PPP, PPPN1, and PPPN2 samples. FTIR spectra were obtained using a Shimadzu IR Affinitu-1 at 4000– 400 cm^−1^ wavenumber interval with 4 cm^−1^ resolution using KBr-method.

### Preparation of PPP, PPPN1 or PPPN2 extracts

The extraction of total polyphenols from PPP, PPPN1, or PPPN2 was performed using a composite design involving five factors. These factors included (i) MeOH concentration (25, 50, 75, and 100%), (ii) extraction time (15, 30, 45, and 60 min), (iii) temperature (20, 40, 60, 80, and 100 °C), (iv) powder-solvent ratio (1/10, 1/8, 1/6, 1/4, and 1/2), and (v) nanoparticle diameters (108 and 347 nm). These factors were considered as the main independent variables affecting the yield of polyphenols. The extracts were subsequently filtered using a Whatman No. 1 filter paper and the total phenolic content was determined.

### Total phenolics content

The spectrophotometer Jenway (Model 6105, UK) was used to determine the total polyphenols in PPP, PPPN1, and PPPN2 extracts using the Folin-Ciocalteu method^19^. The total polyphenols were quantified as mg of gallic acid equivalent per g of extract (mg GAE g^−1^). The standard curve of gallic acid with a correlation coefficient of R^2^ = 0.996, is presented by Eq. (1):

1 $$\:Y=\:0.04+0.01x\:$$

Where x was the phenolics concentration and y was the optical density.

### HPLC-PDA analysis of phenolic acids

Phenolic acids (free, conjugated, and bound) were extracted from PPP or its nanoparticle^20^. A 1 g sample in Erlenmeyer flask was defeated twice on mechanical shaker with hexane (40:1 v/w) for 1 h at 25 °C. The defatted sample on Whatman No. 1 paper was then air-dried in a hood at 25 °C. Subsequently, the defatted sample was then extracted twice with 80% MeOH (50:1 v/w) for 1 h at 25 °C. After each extraction, the mixture was filtered through Whatman No. 1 filter paper. The combined MeOH extracts were then evaporated to dryness using a rotary evaporator at 40 °C. To extraction of free phenolic acids, the obtained residue from evaporation of MeOH extract of each sample was re-dissolved in 10 mL acidified water (pH 2 with 6 M HCl) and partitioned with 30 mL of ethyl ether: ethyl acetate (1:1) in a funnel separator, three times. The organic layers contained free phenolic acids were combined and concentrated to dryness using a rotary evaporator at 40 °C and reconstituted in 2 mL MeOH. To extraction of conjugated phenolic acids, the aquas phase was neutralized to pH 7 with 2 M NaOH and dried using vacuum oven at 50 °C overnight. The residue was dissolved in 10 mL of 2 M NaOH and stirred for 4 h at 25 °C. The solution was then acidified to pH 2 with 6 M HCl and extracted three times with ethyl ether and ethyl acetate (1:1). The resulting organic layers contained conjugated phenolic acids were combined and concentrated to dryness using a rotary evaporator at 40 °C and reconstituted in 2 mL MeOH. To extraction of bound phenolic acids, the obtained residue from evaporation of methanolic extract was alkaline hydrolyzed by 40 mL of 2 M NaOH and stirred for 4 h at 25 °C. The solution was then acidified to pH 2 with 6 M HCl and extracted three times with ethyl ether and ethyl acetate (1:1). The resulting organic layers contained bound phenolic acids were combined and concentrated to dryness using a rotary evaporator at 40 °C and reconstituted in 2 mL MeOH.

HPLC analysis was conducted using an Agilent Technologies 1100 series liquid chromatograph equipped with an autosampler and a diode-array detector (PDA-detector). The analytical column was Agilent Eclipse XDB C18 (150 × 4.6 μm; 5 μm) with a C18 guard column. The mobile phase consisted of acetonitrile and 2% acetic acid in ddH_2_O (v/v) as solvents A and B, respectively. The flow rate was 0.8 mL min^−1^ during the total run time of 70 min at a temperature of 28-±2 °C. The gradient program was as follows: 100% A to 85% B in 30 min, 85% A to 50% B in 20 min and 50% A to 0% B in 5 min. and 0% A to 100% B in 5 min. There were 10 min of post-run for reconditioning. All samples were filtered through a 0.45 μm Acrodisc syringe filter (Gelman Laboratory, MI) before injection. Peaks were identified by congruent retention times and UV spectra and compared with those of the standards^21^.

### Radical scavenging assay

The free radical scavenging activity (RSA) of the extracts PPP, PPPN1, PPPN2, and butylated hydroxytoluene (BHT) solutions was assessed^22^. 10 µL of each examined extract or BHT solution were mixed with 1 mL of methanolic solution of 2,2-diphenyl-1-picrylhydrazyl (DPPH^•^, 0.0374 g/L) in a cuvette. The absorbance was measured at 517 nm using a Jenway spectrophotometer (Model 6105, UK) over a period of 30 min. The RSA of DPPH• was calculated using the following equation:

2 $${\text{RAS}}\;\left( \% \right)=\left\{ {\left( {{\text{A}}\;{\text{control}} - {\text{A}}\;{\text{sample}}} \right)/{\text{A}}\;{\text{control}})} \right\} * 100$$

### Reducing power assay

The determination of RP in the extracts of PPP, PPPN1, PPPN2, and BHT solutions was conducted^23^. In this method, 1 mL of the prepared extract was mixed with 2.5 mL of PBS (pH 6.6) and 2.5 mL of potassium ferric cyanide (1%) in a test tube. The mixture was incubated for 20 min at 50 °C, after which 2.5 mL of trichloroacetic acid (10%) was added. Following this, the mixture was centrifuged at 1537×g for 10 min. The upper layer of the solution was combined with an equal volume of ddH_2_O and 0.5 mL of FeCl_3_ (0.1%). The absorbance of the final solution was measured at 700 nm using a spectrophotometer (Jenway, Model 6105, UK). The RP was expressed as an optical density.

### Ferric reducing antioxidant power assay

The assessment of FRAP in the extracts of PPP, PPPN1, PPPN2, and BHT solution was conducted^24^. For the preparation of the FRAP reagent, we mixed a solution of 2,4,6-tripyridyl-s-triazine (TPTZ, 0.312 g/100 mL ddH_2_O), ferric chloride hexahydrate (FeCl_3_·6H_2_O) (0.54 g/100 mL ddH_2_O), and sodium acetate trihydrate (0.272 g/100 mL ddH_2_O) in a ratio of 1:1:10, respectively. The resulting reagent (1500 µL) was combined with a 50 µL of the prepared extract solution in a cuvette. Subsequently, the FRAP values were determined by measuring the optical density at 593 nm using a spectrophotometer (Jenway, Model 6105, UK).

### Statistical analysis

Table 1 presents the symbols and levels of independent variables affected on the total phenolics extraction yield as dependent variable (z) for Box-Behnken design. The identified variable was the total phenolic yield, and the independent variables were MeOH concentration (C), extraction time (t), temperature (T), powder-solvent ratio (R), and nanoparticle diameter (D). Predicting the total phenolics yield (Y) was assumed for each pair of independent variables using a cubic polynomial regression model (Eq. 3^25^).

**Table 1:** Symbols and levels of independent variables affected the total phenolics extraction yield as dependent variable (z) for the Box-Behnken design.

Independent variables (X or y)	Symbol	Levels				
Methanol concentration (%)	C	25	50	75	100	
Extraction time (min)	t	15	30	45	60	
Extraction temperature	T	20	40	60	80	100
Powder-solvent ratio (%)	R	1:10 (10%)	1:8 (12.5%)	1:6 (16.7%)	1:4 (25%)	1:2 (50%)
Nanoparticle diameters	D	125	373			

3 $$\:Y=\:{\beta\:}_{0}+\sum\:^{2}{\beta\:}_{1}{X}_{i}^{}+\:\sum\:^{2}{\beta\:}_{2}{X}_{i}^{2}\:+\:\sum\:^{2}{\beta\:}_{3}{X}_{i}^{3}+\:\sum\:^{2}{\beta\:}_{4}{X}_{i}^{}{X}_{j}^{}\:\:$$

Predicting total phenolics yield (Y) was assumed to be 5 independent variables using a quintic polynomial regression model (Eq. 4). The PROC REG procedure of the Statistical Analysis System was used^26^.

4 $$\: \begin{aligned} Y = & \beta _{0} + \sum {^{5} } \beta _{1} X_{i} + \sum {^{4} } \beta _{2} X_{i}^{2} + \sum {^{4} } \beta _{3} X_{i}^{2} X_{j}^{2} + \sum {^{4} } \beta _{4} X_{i}^{3} \\ & + \sum {^{4} } \beta _{5} X_{i}^{3} X_{k}^{3} + \sum {^{2} } \beta _{6} X_{i}^{4} + \sum {^{4} } \beta _{7} X_{i}^{4} X_{l}^{4} + \sum {^{3} } \beta _{8} X_{i}^{5} X_{m}^{5} \\ \end{aligned}$$

In the equations, *β*_*0*_, *β*_*1*_, *β*_*3*_, *β*_*4*_, *β*_*5*_, *β*_*6*,_ *β*_*7*,_ and *β*_*8*_ represent the intercept, linear, quadratic, cubic, quartic, and quintic interaction regression coefficient terms, respectively. X_i_, X_j_, X_k_, X_l_, and X_m_ are independent variables. ANOVA analysis was performed using the PROC ANOVA procedure of the Statistical Analysis System^26^. Duncan’s multiple ranges were used at a significance level of 5% to compare means^27^. Results with different alphabetical letters indicate significant differences. Unless indicated, all analysis were performed in triplicate, and the results were presented as mean ± standard deviation (SD).

## Results and discussion

### The particle size and micrographs of PPPN1 and PPPN2

**Fig. 1:**
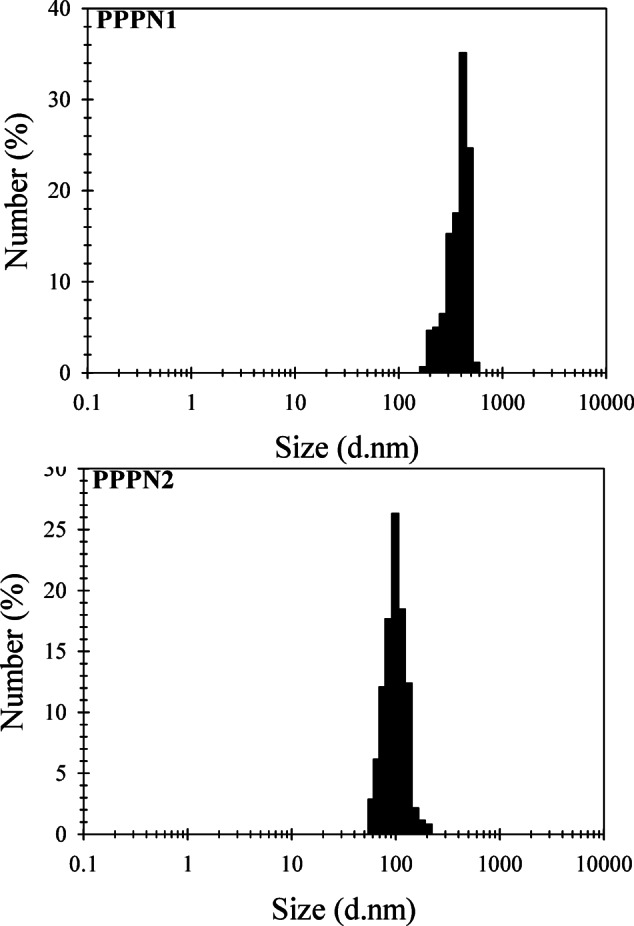
Diameters fractionation of PPPN1 and PPPN2 particles with average diameter sizes of 347 and 112 nm, respectively.

Figure 1 provides a detailed particle size fractionation for PPPN1 and PPPN2. It presents the distribution and relative abundance of particles at different diameter ranges. PPPN1 exhibited a gradual increase in particle size, starting from 164 nm with a percentage of 0.7%. It then reached its largest diameter at 396 nm, with a significantly higher percentage of 35.2%. The particle diameter increased progressively, while the percentage declined until it reached its maximum diameter of 615 nm with a percentage value of 0.8%. PPPN2 began with a percentage of 2.8% at a diameter of 56 nm and gradually increased to a maximum diameter of 95 nm with a percentage of 26.3%. Following that, the particle sizes continued to increase incrementally, but the percentage gradually decreased to 0.8% until reaching the maximum diameter of 198 nm. Notably, PPPN1 exhibits larger particle sizes compared to PPPN2, with average diameters of 347 and 112 nm, respectively.

**Fig. 2:**
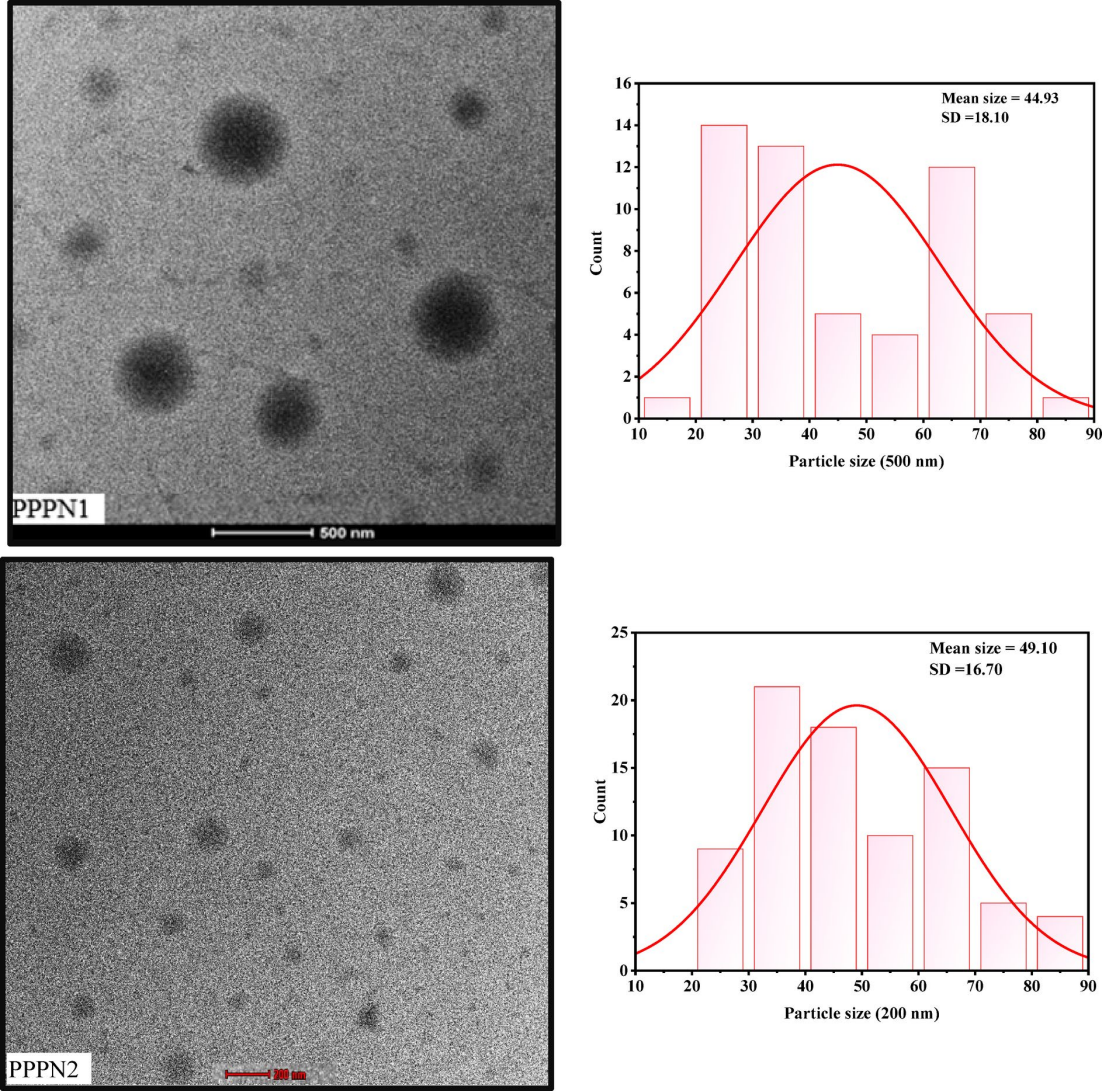
TEM micrographs of PPPN1 and PPPN2 and their histogram analysis.

Figure 2 showcases TEM micrographs of PPPN1 and PPPN2. The surface morphology of PPPN1 and PPPN2 fractions appeared spherical in shape. The particle sizes ranged from 164 to 615 nm and 56 to 198 nm, respectively. These findings indicate that both fractions fall within the nanoscale range. The application of this approach proves its applicability to produce nano powder. In our earlier work, we attempted to increase the extractability of phenolics from nano-pomegranate fractions with average diameters ranging from 125 to 373 nm. The importance of nanoparticles in increasing polyphenol absorption and bioavailability has already been recognized^28^.

### Total polyphenols

**Fig. 3:**
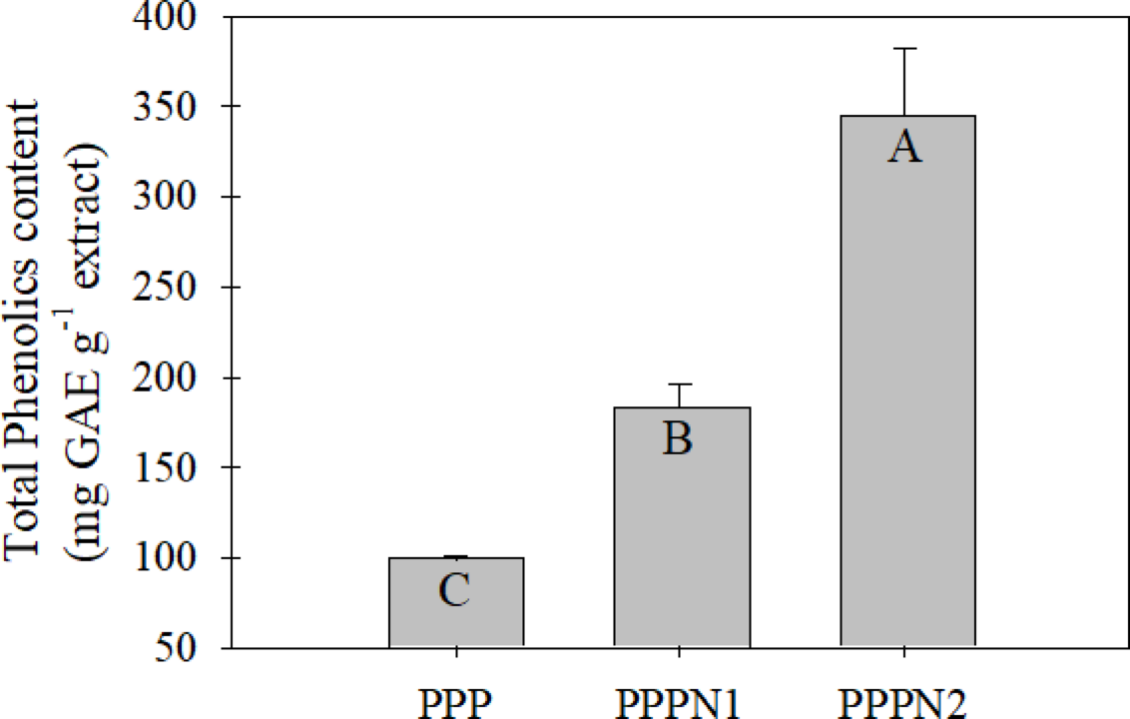
Total phenolics content (mg GAE/g extract) extractability of PPP, PPPN1, and PPPN2 at constant conditions as MeOH concentration = 75%, extraction time = 30 min, extraction temperature = 60 °C, and powder-solvent ratio = 1:6 (16.7%). Different capital alphabets are significantly different (*P* < 0.05). The results were presented as mean ± standard deviation (SD).

Figure 3 displays the total phenolic content of three nano-fractions PPP, PPPN1, and PPPN2. The extraction process was conducted under constant conditions, including a 75% MeOH concentration, 30 min of extraction time, 60 °C extraction temperature, and a powder-solvent ratio of 1:6 (16.7%). The study revealed that both PPPN1 and PPPN2, as nano-fractions, exhibited significantly higher total phenolic content (*P* < 0.05) compared to PPP. The determined values for PPPN1 and PPPN2 were 182 and 344 mg GAE g^−1^, respectively, while PPP showed a lower total phenolic content of 99 mg GAE g^−1^. Consequently, both PPPN1 and PPPN2 were selected for further modelling experiments to determine the optimal conditions for extracting total phenolics from pomegranate peel. During a previous study, we conducted an initial investigation focusing on the efficacy of nano-fractions derived from pomegranate peels^3^. These nano-fractions had particle diameters ranging from 125 to 373 nm and exhibited remarkable effectiveness in significantly enhancing the extraction of total phenolics. To improve polyphenol extraction from PPP, a variety of approaches were used, including ultrasonic waves^16,29^ and various solvents^17^. The nano-fractions generated in this study (PPPN1 and PPPN2) improved polyphenol extractability compared to earlier investigations. According to the research, lowering particle size to the nanoscale boosted their surface area. This increased surface area allowed polyphenol extraction.

### FTIR analysis

**Fig. 4:**
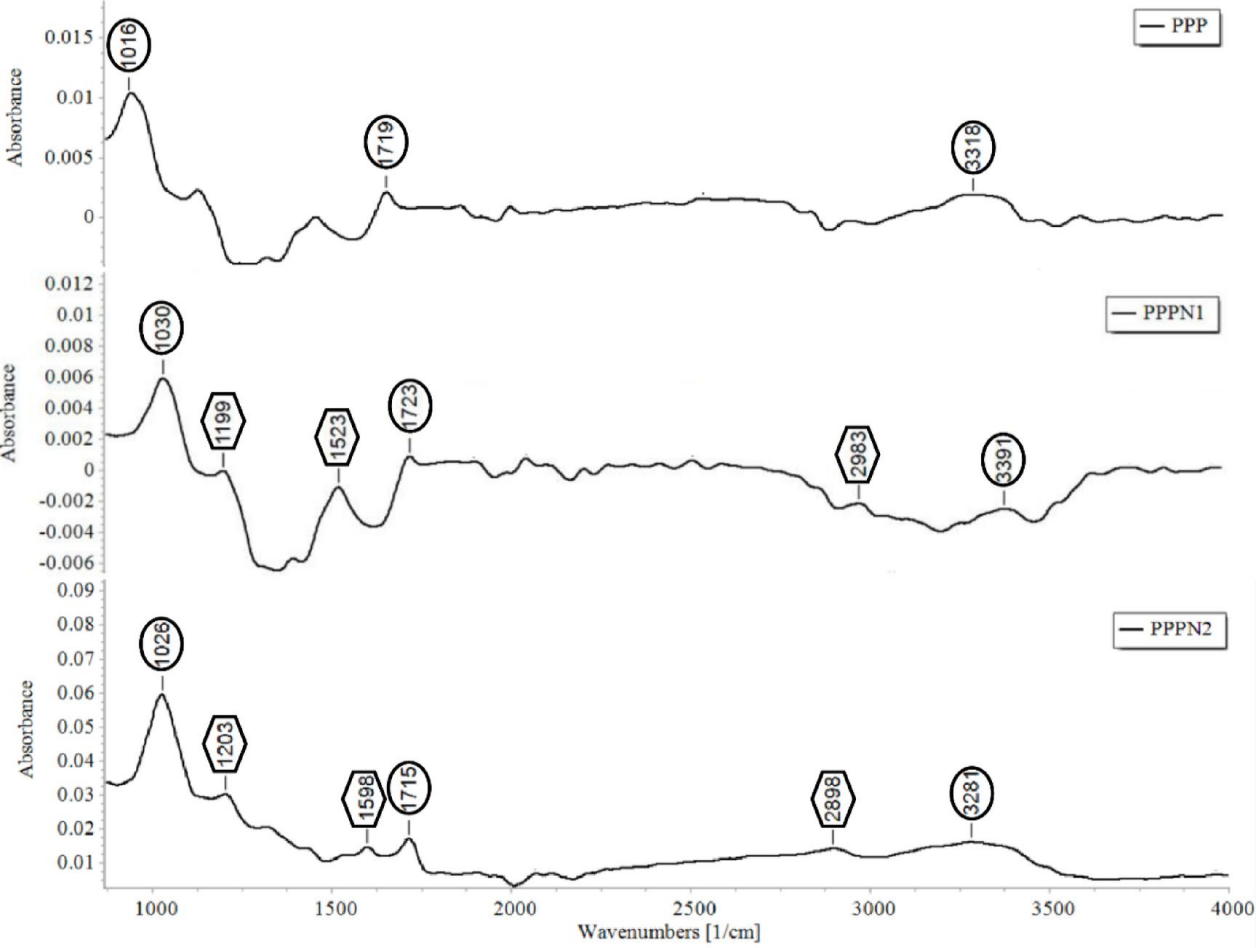
Fourier-transform infrared spectra of PPP and its nanoparticles highlighting their key functional peaks.

The FTIR spectra of PPP, PPPN1, and PPPN2 were utilized to evaluate alterations in the functional groups present on their surfaces due to the grinding process to the nanoscale. As illustrated in Fig. 4, FTIR spectra of PPP and two nano-fractions displayed several absorption peaks at wavenumber ranging from 400 to 4000 cm^−1^. The observation of peaks at wavenumber ranging between 1016 and 1203 cm^−1^ referred to the bending vibration of the presence of some aliphatic and aromatic functional groups of polyphenols including C–O and C=O^30^. The spectra of PPPN1 and PPPN2 showed characteristic peaks at 1523 and 1598 cm^−1^ corresponding to occurrence of amide II (attributed to N–H bending and C–N stretching vibrations, orderly)^5^. The bands situated at wavenumber ranging between 1715 and 1723 cm^−1^ are described as C=O groups for carboxylic acid, acetate groups COO, ketone, and aldehyde^31^. At wavenumber 2983 and 2898 cm^−1^, the bands are referred as C-H stretching of methyl and methoxy groups, alongside methyl and methylene groups from carboxylic acids^10^. The broad band at absorption ranged between 3281 and 3391 cm^−1^ is attributed to the O-H stretching of polyphenols^5^. With an increase in the surface area of particles, the intensity of absorption for the spectra peaks of PPPN2 was higher compared to those of PPP and PPPN1. This phenomenon indicates a greater concentration of detectable compounds. Moreover, spectra of PPPN1 and PPPN2 exhibited peaks ranging from wavenumber 1199 to 1598 cm^−1^ and 2898 to 2983 cm^−1^, which were absent in the PPP spectra. This shows that chemicals are more accessible on the particle surface, demonstrating the efficiency of the nano-pomegranate peel formulation. Polyphenols’ enhanced absorbance also makes subsequent extraction simpler.

### The role of five studied independent variables on phenolic extraction yield

**Fig. 5:**
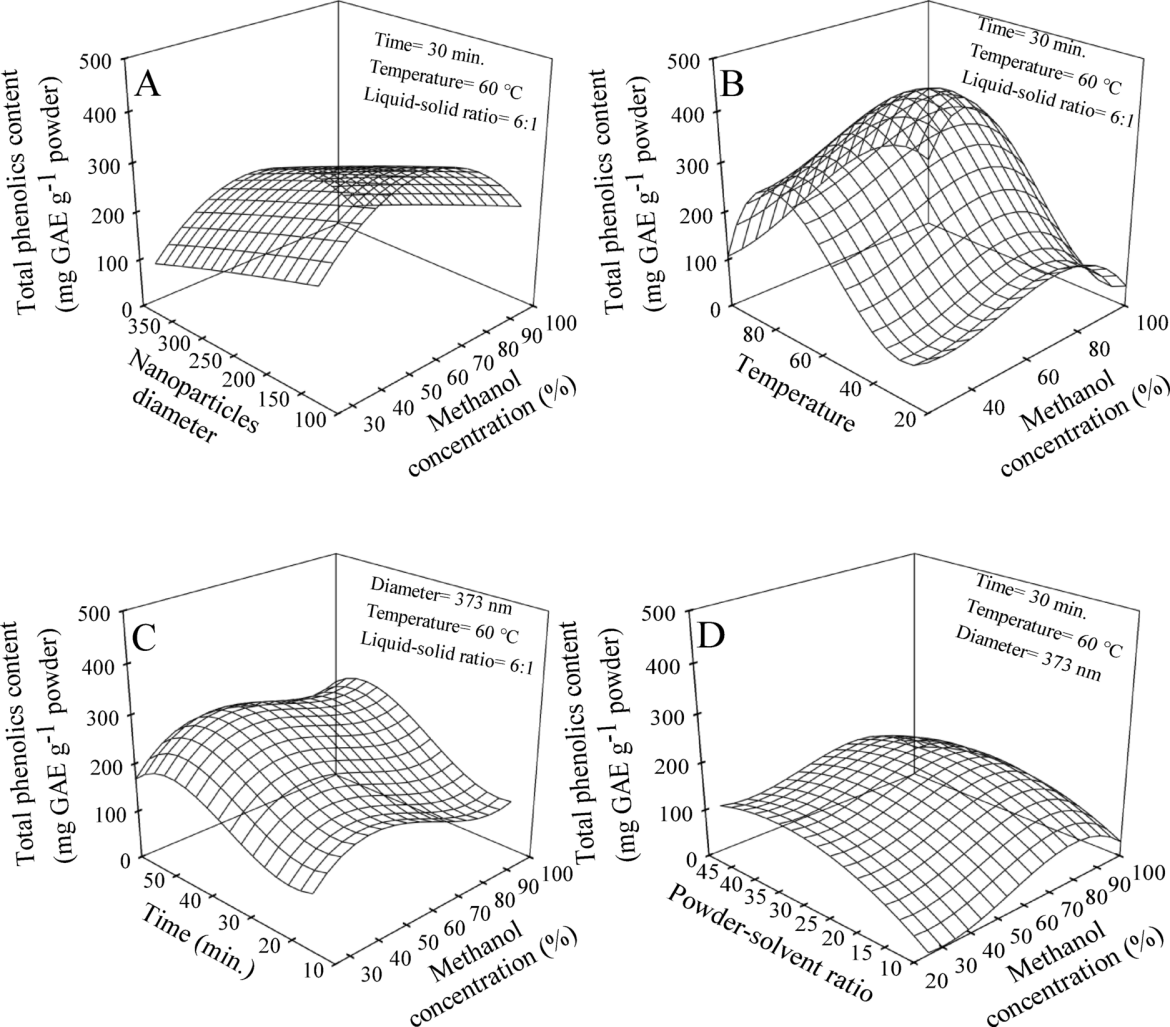
Response surface of total phenolics extraction yield affected by methanol concentrations versus nanoparticles diameter (**A**), temperature (**B**), time (**C**), and powder-solvent ratio (**D**).

The 3D-response surface method examined the effect of the interaction between two independent variables on the total polyphenols. For each pair of independent variables, the analysis was conducted while keeping the other three independent variables at constant values. The effects of MeOH concentrations, extraction time, temperatures, powder-solvent ratios, and nanoparticle diameters on the extraction yield of polyphenols were illustrated as a 3D-response surface portrayed. It was indicated that the phenolics extraction yield increased as the MeOH concentration increased, and the particle diameter decreased (Fig. 5A). The highest predicted yield (Eq. 5) of 350 mg GAE g^−1^ was observed when the MeOH concentration and particle diameter were 65.5% and 125 nm, respectively. The polynomial regression analysis of the phenolics extraction yield considering varying MeOH concentrations and temperatures is depicted in Fig. 5B. The observed changes in the extraction yield highlight the influence of both independent variables (Eq. 6). Notably, the highest predicted phenolics yield of 381 mg GAE g^−1^ was achieved using a MeOH concentration of 76.5% and a temperature of 80 °C. The impact of MeOH concentrations and extraction time on the phenolics yield was examined using a response surface (Fig. 5C). Both MeOH concentrations and time were significantly influenced by the phenolics yield (Eq. 7). Based on the predicted values of the variables studied, the highest phenolics yield of 245 mg GAE g^−1^ was obtained at a MeOH concentration of 76.5% and a time of 51.5 min. The 3D-response surface plot depicted in Fig. 5D illustrates the relationships between the phenolics yield and both MeOH concentrations and powder-solvent ratio. The phenolics yield exhibited an increase when the MeOH concentration ranged from 25 to 76.5% and the powder-solvent ratio ranged from 10 to 35% reaching its highest predicted value of 167.1 mg GAE g^−1^ (Eq. 8). Conversely, a decrease in yield was observed as both MeOH concentration and powder-solvent ratio increased to 100 and 50%, respectively.

**Fig. 6:**
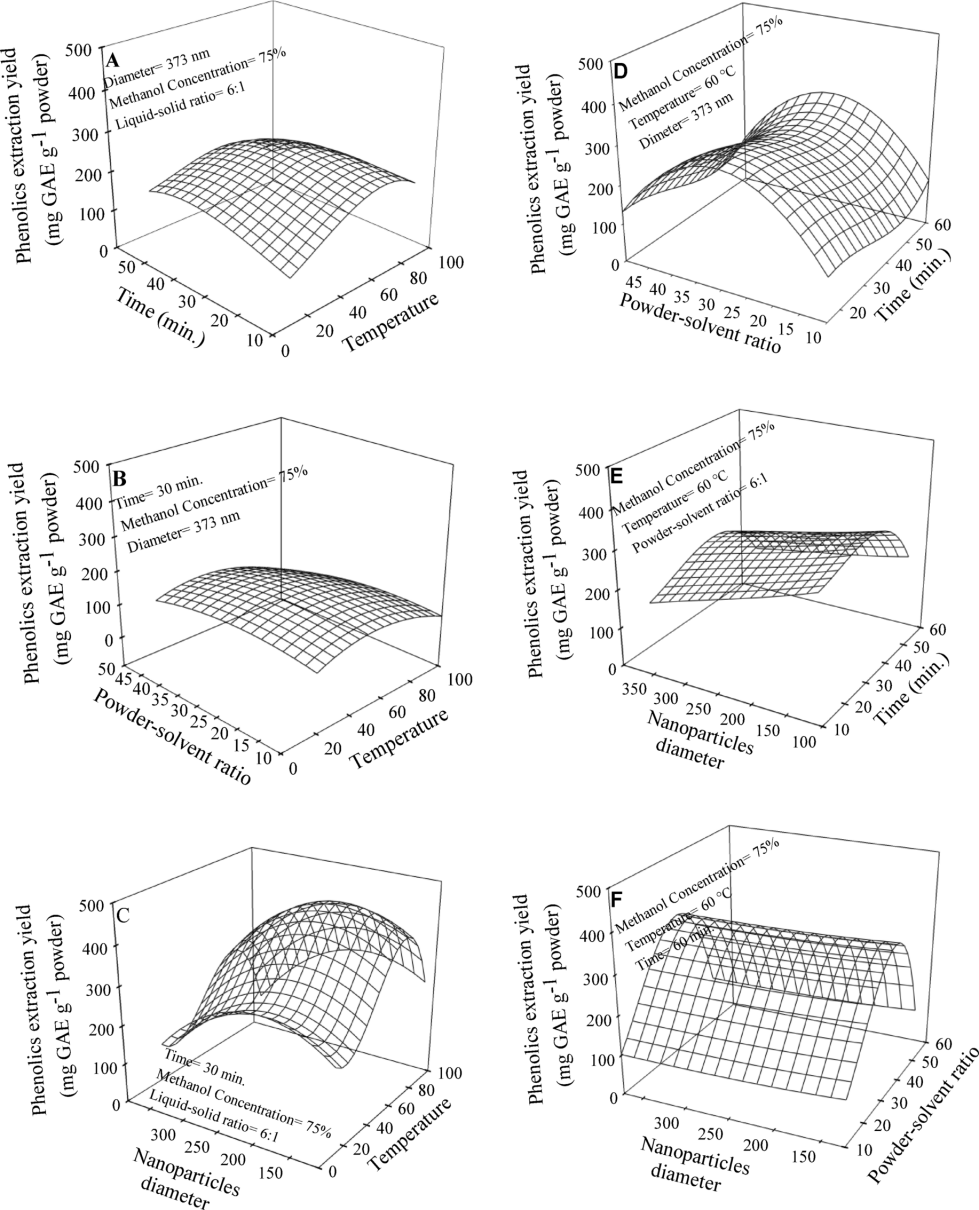
Response surface of total phenolics extraction yield affected by temperature versus time (**A**), powder-solvent ratio (**B**) and nanoparticles diameter (**C**); time versus powder-solvent ratio (**D**), nanoparticles diameter (**E**) as well as, powder-solvent ratio versus nanoparticles diameter (**F**).

The impact of temperature and extraction time on the extracted yield of phenolics obtained was also analyzed (Fig. 6A). According to the regression analysis (Eq. 9), the highest predicted extraction yield was 205.6 mg GAE g^−1^ at a temperature of 75 °C and an extraction duration of 37.5 min. The relationship between the extraction temperature and the powder-solvent ratio on the quantity of obtained phenolics was examined (Fig. 6B). Increasing both temperature (from 0 to 50 °C) and powder-solvent ratio (from 10 to 25%) increased the quantity of extracted phenolics. The regression analysis (Eq. 10) predicted the highest yield of phenolics to be 139.5 mg GAE g^−1^ at a temperature of 50 °C and a powder-solvent ratio of 25%. However, further increases in these factors resulted in a decrease in the quantity of extracted phenolics. The effect of extraction temperature and nanoparticle diameter on the extractability of phenolics is presented in Fig. 6C. The response surface of 3D-plot (Eq. 11) revealed that increasing the extraction temperature improved the extraction efficiency reaching a maximum limit at 75 °C. The response surface for nanoparticle diameter demonstrated that the optimal extraction occurred at a diameter of 140 nm. The regression analysis predicted a phenolics yield of 406 mg GAE g^−1^. The relationship between extraction time and powder-solvent ratio was investigated in terms of their impact on the extraction yield of phenolics (Fig. 6D). The regression analysis (Eq. 12) demonstrated the significant influence of both variables on the extraction process. Notably, it was observed that an extraction time of 30 min, yielding the highest quantity of phenolics. Furthermore, increasing the powder-solvent ratio led to the highest extraction yield of phenolics at a ratio of 17.8%. Based on the regression analysis using the specified values for extraction time and powder-solvent ratio, the predicted quantity of extracted phenolics was 305 mg GAE g^−1^. The impact of extraction time and nanoparticle diameter on the phenolics extractability is illustrated in Fig. 6E. The regression analysis (Eq. 13) indicated that both variables had a significant effect on the extraction process. The extraction time of 37.5 min was identified as the expected duration that yielded the highest quantity of extraction. Furthermore, it was observed that a decrease in nanoparticle diameter increased the phenolics yield, with the highest extraction observed at a diameter of 112 nm. According to the specified values for extraction time and nanoparticle diameter, the predicted phenolics yield was 328 mg GAE g^−1^. Figure 6F investigated the influence of powder-solvent ratio and nanoparticle diameter on the extraction phenolics yield. The regression analysis (Eq. 14) revealed that the powder-solvent ratio of 35% was identified as the optimal extraction ratio. Furthermore, a decrease in nanoparticle diameter was observed to correlate with an increase in phenolics extractability. The highest extraction was observed at a diameter of 112 nm. The highest predicted quantity of extracted phenolics was 365 mg GAE g^−1^ at the identified values of powder-solvent ratio and nanoparticle diameter. The quantity of extracted phenolics from pomegranate peels varies depending on the type of solvent used. In this study, we employed MeOH to optimize the phenolics extraction process based on previous studies that demonstrated its high efficiency in extracting polyphenols from pomegranate peels^3,6^. The solvent polarity is a critical factor influencing the quantity of extracted phenolic compounds^17^. Additionally, the extraction efficiency of phenolics depends on s extraction conditions such as the as time, temperature, particle diameter, and the solvent-to-powder ratio^12,32^.

Table 2 presents the regression coefficients for the interaction between pairs of independent variables while holding the other three variables constant. The regression analysis aimed to establish cubic polynomial models for the yield of extracted phenolic compounds. The adequacy of the models was assessed using an analysis of variance, which indicated that the regression models were highly significant (*P* < 0.05). The correlation coefficients of different models ranged from 0.776 to 0.994, indicating a strong relationship among the variables. The predicted models obtained from the regression analysis can be used to identify the optimal conditions necessary for achieving a high yield of extracted phenolics. The optimum predicted values for MeOH concentration, time, temperatures, powder-solvent ratios, and nanoparticle diameters fell within the ranges of 65.5 to 76.5%, 30 to 51.5 min, 50 to 80 °C, 17.8 to 35%, and 112 to 140 nm, respectively. By comparing the models, it can be concluded that these conditions resulted in a high yield of polyphenols ranging between 167.1 and 406 mg GAE g^−1^. In summary, the regression analysis provided insights into the interaction effects among the independent variables and allowed for the prediction of optimal extraction conditions to maximize the yield of phenolic compounds. These findings can be valuable in enhancing the extraction process and obtaining high quantity phenolics. The conditions or methods employed during extraction have an impact on the amount and composition of polyphenols. Specifically, the polarity of the solvent used for extraction plays a crucial role in determining these factors^3,33^.

**Table 2:** Regression coefficients of cubic polynomial model for total phenolics extraction yield response surface (z).

Equation number	Independent variables (X × y)	Parameters								*R* ^2^
		Linear			Quadratic		Cubic		Interaction	
		β_0_	β_1_	β_2_	β_3_	β_4_	β_5_	β_6_	β_7_	
5	C × D	39.8	12.08	-0.39	-0.099	1.7e-05	7.1e-06	-2.9e-07	-0.001	0.931
6	C × T	515.8	-9.37	-25.44	0.213	0.593	-0.001	-0.004	0.017	0.807
7	C × t	-22.2	19.50	-21.45	-0.322	0.737	0.002	-0.007	0.020	0.791
8	C × R	17.0	-8.66	11.37	0.205	-0.137	-0.001	-9.8e-05	-0.020	0.850
9	T × t	-6.5	1.35	1.93	0.051	0.078	-4.8e-04	-0.001	-0.031	0.776
10	T × R	-11.2	3.99	4.15	-0.041	-0.066	6.2e-05	-9.1e-04	-0.013	0.779
11	T × D	120.6	-20.00	3.28	0.509	-0.007	-0.003	-2.2e-07	-0.004	0.742
12	t × R	-210.6	12.20	27.13	-0.447	-0.432	0.005	-2.1e-04	-0.009	0.802
13	t × D	425.7	-6.24	-1.05	0.321	0.002	-0.004	-2.0e-06	0.006	0.855
14	D × R	84.9	0.22	-0.36	0.763	9.2e-04	-0.015	-7.6e-07	-1.8e-04	0.994

R^2^ = Correlation coefficient.

The 3D-response surface studies expressed the interaction effects between each two independent variables (Table 2). Thus, interaction among all independent variables is necessary for enhancing the polyphenols’ extractability. Multiple regression coefficients were presented in Eq. (15) to predict a quintic model to optimize the different independent variables. The model was tested for adequacy by analysis of variance. The regression model for data was highly significant (*P* < 0.05) with R^2^ = 0.7485. The predicted model for the phenolics yield (Y) was reported as follows:

15 $$\begin{aligned} y = & 573.7 - 23.2~C - 13.6t + 23.7T - 49.97R - 0.084D + 0.54C^{2} \\ & + 0.414t^{2} - 0.762T^{2} + 4.43R^{2} - 2.6 \times 10^{{ - 5}} C^{2} t^{2} - 5.5 \times 10^{{ - 6}} C^{2} T^{2} \\ & - 4.8 \times 10^{{ - 5}} C^{2} R^{2} + 2.37 \times 10^{{ - 7}} C^{2} D^{2} - 0.003C^{3} - 0.004t^{3} + 0.01T^{3} - 0.151R^{3} \\ & + 1.51 \times 10^{{ - 8}} C^{3} t^{3} + 2.34 \times 10^{{ - 9}} C^{3} T^{3} + 3.36 \times 10^{{ - 8}} C^{3} R^{3} - 2.35 \times 10^{{ - 11}} C^{3} D^{3} \\ & - 4.5 \times 10^{{ - 5}} T^{4} + 0.002R^{4} - 3.18 \times 10^{{ - 12}} C^{4} t^{4} - 3.41 \times 10^{{ - 13}} C^{4} T^{4} - 8.5 \times 10^{{ - 12}} C^{4} R^{4} \\ & + 4.49 \times 10^{{ - 16}} C^{4} D^{4} + 2.25 \times 10^{{ - 16}} C^{5} t^{5} + 1.61 \times 10^{{ - 17}} C^{5} T^{5} + 7.26 \times 10^{{ - 16}} C^{5} R^{5} \\ \end{aligned}$$

Consequently, the obtained predicted model (Eq. 15) makes it possible to identify the optimum conditions required to produce a high phenolics yield. It could be concluded that the highest predicted phenolics yield was 406 mg GAE g^−1^ at estimated different independent variables were 75%, 45 min, 80 °C, 16.7% and 112 nm for MeOH concentrations, extraction time, temperatures, powder-solvent ratios, and nanoparticles diameters, respectively. The obtained verified phenolics yield was 394.6 mg GAE g^−1^ with an application of the previous model parameters. The results obtained from Eq. (15) appear promising as the total extracted phenolic value (406 mg GAE g^−1^) surpassed that of several previous studies. Specifically, El-Hadary and Taha^6^ reported a methanolic extract of 90 mg GAE g^−1^, while Kazemi, et al.^29^ achieved 320.2 mg GAE g^−1^ by utilizing ultrasound assistance during the extraction process. Similarly, Živković, et al.^16^ employed ultrasound and obtained the highest extraction of 442.48 mg GAE g^−1^ extract (88.4 mg GAE g^−1^). Additionally, Derakhshan, et al.^34^ obtained the highest total phenolic value of 361 mg GAE g^−1^. These findings indicate that the optimized extraction process for total phenolics holds promise and is applicable.

### Phenolic acids profile of PPP, PPPN1, and PPPN2

The phenolic acids profile of the prepared PPP, PPPN1, and PPPN2 extracts at the optimum predicted conditions (Eq. 15) was determined. The compounds are classified into three groups: Free, conjugated, and bound phenolic acids (Table 3). PPP contained the lowest concentrations of phenolic acids among all the compounds in each group, while PPPN2 demonstrated the highest concentrations of phenolic acids. Among these, gallic, chlorogenic, and vanillic acids have peaked as free phenolic acids with values of 24.26 to 32.07, 10.79 to 17.74, and 4.28 to 4.49 mg g^−1^, respectively. The same trends were observed with conjugated and bound polyphenols profile with differentiation in the compound types. Moving to the conjugated fraction, the compounds with the highest concentrations are rosmarinic and ellagic acids ranging from 2.68 to 2.21 and 1.48 to 2.41 mg g^−1^, respectively. At the same time, the bound fractions that had the highest concentrations were gallic, rosmarinic, and protocatechuic. The concentrations observed ranged from 73.57 to 96.13, 2.32 to 5.39 and 1.59 to 4.48 mg g^−1^, respectively. It was observed that decreasing the particle diameter increased the concentration of detected phenolic acid compounds. Particularly, PPPN2 exhibited the highest concentration of total free, conjugated, and bound polyphenols (65.09, 27.68, and 111.45 mg g^−1^, respectively), indicating enhanced extractability. Pomegranates contain significant amounts of phenolic acids, such as gallic and ellagic acids belonging to the hydroxybenzoic acid group. Where, caffeic, chlorogenic, and p-coumaric acids belong to the hydroxycinnamic acid group^35^. In a previous study, authors found that pomegranate peels are an economical source of biologically active polyphenols^8,36^. They presented several polyphenols that align with the compounds defined in our study. However, further studies regarding isolation, identification and a comprehensive characterization of the activity are needed.

**Table 3:** Phenolic acids profile (mg g^[-1^) of PPP and its nanoparticles extracts are prepared using the optimal predicted independent variables.

Compounds	Free			Conjugated			Bound		
	PPP	PPPN1	PPPN2	PPP	PPPN1	PPPN2	PPP	PPPN1	PPPN2
Gallic	24.26	28.43	32.07	0.42	0.66	7.38	73.57	85.77	96.13
Protocatechuic	2.06	2.26	2.42	1.76	2.08	3.11	1.59	2.25	4.48
Chlorogenic	10.79	14.87	17.74	ND	ND	ND	ND	ND	ND
Vanillic	4.28	4.31	4.49	0.41	0.49	0.52	0.24	0.63	0.92
Syringic	0.42	0.56	0.93	0.80	0.88	0.95	0.34	0.43	0.54
Caffeic	0.35	0.31	0.32	ND	ND	ND	0.15	0.10	0.19
Ellagic	0.31	0.38	0.50	1.48	1.75	2.41	1.74	2.30	2.94
Ferulic	0.28	0.33	0.36	0.35	0.43	0.44	0.22	0.21	0.25
Cinnamic	0.04	0.03	0.03	0.37	0.39	0.44	ND	0.24	0.34
Sinapic	0.34	0.40	0.43	0.60	0.82	0.98	ND	ND	ND
Rosmarinic	0.24	0.32	0.35	2.68	2.38	2.21	2.32	4.27	5.39
Total	**43.37**	**52.2**	**59.64**	**8.87**	**9.88**	**18.44**	**80.17**	**96.2**	**111.18**

*ND* not detected.

### Antioxidant activity of nano-PPP verified extract

The quintic polynomial regression model was used to determine the predicted optimal conditions given the highest phenolics yield from PPPN2. The highest yield (406 mg GAE g^−1^) was achieved at 75% °C, 45 min., 80 °C, and 16.7%. The extract of PPPN2 contained 4.06 mg GAE g^−1^. The antioxidant activities were evaluated using different assays, namely RSA, RP, and FRAP (Table 4). The results indicate that the PPPN2 extract exhibited the highest RSA (93.7%), with no significant differences (*P* < 0.05) compared to BHT solutions at concentrations of 150 or 200 ppm. Similarly, the RP of PPPN2 was significantly higher (0.974) compared to BHT solutions at 150 or 200 ppm to be 0.883 and 1.052, respectively, without significant differences (*P* < 0.05). Regarding FRAP, PPPN2 displayed a superior antioxidant activity to be 1.375, which was comparable to BHT-values at 150 and 200 ppm, with values of 1.237 and 1.554, respectively. Reductones, derived from polyphenols, have antiradical properties that allow them to donate hydrogen atoms and inhibit free radicals^37,38^. They also react with radicals produced during peroxidation, preventing formation of peroxide. Furthermore, in the Fenton reaction, the reduction of Fe^2+^ helps protect against oxidative damage^39^. The statistical analysis of variance confirmed the significance (*P* < 0.05) of these parameters. The correlation coefficients ranged between 0.867 and 0.911, indicating strong positive relationships between the antioxidant activities of the PPPN2 extract and the effects of BHT solutions. The pomegranate peel contains various bioactive substances, such as quercetin, catechin, phenolic acids, and other antioxidative polyphenols^40^ that play a key role in managing oxidative stress^38,41^. These findings demonstrate the strong antioxidant properties of the nano-PPP extract prepared using our predicted conditions that might have potential applications in industries requiring antioxidant properties for various purposes^42^. In a study by El-Hadary and Ramadan^20^, it was observed that the methanolic extract of pomegranate exhibited a strong antioxidant activity, as demonstrated by high DPPH (93.7%). The superior antioxidant effect observed in the nano-fraction of pomegranate peel agreed with our FTIR findings (Fig. 4). We found that smaller particle sizes of pomegranate peel increased absorption of active groups, facilitating easier extraction of polyphenols. The spectra of PPPN2 showed a higher variety and quantity of phenolics. This increase in phenolic concentrations correlates positively with the observed antioxidant effects (Table 3).

**Table 4:** Antioxidant activities of verified extract prepared using predictive conditions in comparison with different concentrations of BHT solutions using three tested methods.

Treatments	RSA (DPPH^·^)	RP	FRAP
PPPN2	93.7^a^ ± 1.45	0.974^ab^ ± 0.09	1.375^ab^ ± 0.24
BHT_50 ppm_	67.7^c^ ± 9.96	0.633^d^ ± 0.04	0.655^d^ ± 0.12
BHT_100 ppm_	80.2^b^ ± 0.87	0.800^c^ ± 0.03	0.967^c^ ± 0.11
BHT_150 ppm_	88.1^ab^ ± 0.81	0.883^bc^ ± 0.02	1.237^bc^ ± 0.04
BHT_200 ppm_	94.3^a^ ± 2.33	1.052^a^ ± 0.06	1.554^a^ ± 0.18
R^2^	0.872	0.911	0.867

Means in the same column with different uppercase letters are significantly different (*P* ≤ 0.05).

Values are mean (*n* = 3) ± standard deviations.

R^2^ = Correlation coefficient.

## Conclusions

This study advances sustainable agro-waste valorization by optimizing polyphenol extraction from pomegranate peel through synergistic integration of conventional parameters (75% methanol, 45 min, 80 °C, 16.7% solvent ratio) and nanoparticle modulation. Strategic nanonization to 112–347 nm fractions enhanced bio-accessibility, with PPPN2 (112 nm) exhibiting superior phenolic enrichment (406 mg GAE/g) versus bulk or coarser nano-fractions. FTIR confirmed nano-specific spectral signatures, including intensified C–O phenolic acid (1016–1203 cm^−1^) and O–H hydroxyl (3281–3391 cm^−1^) bands, correlating with liberated bound phenolics and elevated antioxidant efficacy (DPPH, ABTS, FRAP). The quintic regression model validated nanoscale-driven yield optimization while preserving bioactivity which is a critical milestone for industrial adoption. These nano-fractions represent a scalable, cost-effective nutraceutical ingredient aligning with circular bioeconomy principles. Future directions require pilot-scale process validation and bioavailability assessments to translate this waste-to-value strategy into commercial functional foods^43^.

## Data Availability

Data sets generated during the current study are available from the corresponding author on reasonable request.
